# Supplementary material for: Antibiotic tolerance is associated with a broad and complex transcriptional response in E. coli
Source: Sci Rep. 2021 Mar 17;11:6112. doi: 10.1038/s41598-021-85509-7 (PMC7969968; doi:10.1038/s41598-021-85509-7)
Supplement: Supplementary file 4 — Supplementary Information 4. [file 41598_2021_85509_MOESM4_ESM.docx]

**Antibiotic tolerance is associated with a broad and complex transcriptional response** **in *E. coli***

Heather S. Deter^1^, Tahmina Hossain^2^ and Nicholas C. Butzin*^2^

^1^ Department of Bioengineering, University of Illinois at Urbana-Champaign, Urbana, IL, 61801, USA

^2^ Department of Biology and Microbiology. South Dakota State University. Brookings, SD. 57006. USA.

*Correspondence: nicholas.butzin@gmail.com

**Supplemental information**

File S1: Tab-delimited file containing direct regulatory information for each gene according to the Ecocyc^3^ database as of February 2020.

File S2: Tab-delimited file containing regulatory interactions used to make network in Fig. 2A

File S3: Tab-delimited file containing regulatory interactions used to make network in Fig. 3A
